# Regulating the production and biological function of small extracellular vesicles: current strategies, applications and prospects

**DOI:** 10.1186/s12951-021-01171-1

**Published:** 2021-12-14

**Authors:** Lei Luo, Zhi Wu, Yang Wang, Haiyan Li

**Affiliations:** 1 Institute of Microsurgery on Extremities, Shanghai Jiao Tong University Affiliated Sixth People’s Hospital, 600 Yishan Road, Shanghai, 200233 China; 2 School of Biomedical Engineering, Shanghai Jiao Tong University, 1954 Huashan Road, Shanghai, 200030 China; 3 Chemical and Environmental Engineering Department, School of Engineering, RMIT University, 124 La Trobe St, Melbourne, VIC 3001 Australia

**Keywords:** Small extracellular vesicles (sEVs), Tissue repair and regeneration, sEVs yield, Desired biological function

## Abstract

Numerous studies have confirmed the great application potentials of small extracellular vesicles (sEVs) in biological medical field, especially in tissue repair and regeneration. However, the production capability of sEVs by noncancerous cells is very limited, while their dosage requirements in disease treatments are usually very high. Meanwhile, as cell aging, the sEV production capability of cells decreases and the biological function of sEVs changes accordingly. In addition, for special applications, sEVs carrying desired bioactive substances should be designed to perform their expected biological function. Therefore, improving the production of sEVs and precisely regulating their biological function are of great significance for promoting the clinical applications of sEVs. In this review, some of the current classic strategies in affecting the cellular behaviors of donor cells and subsequently regulating the production and biological function of their sEVs are summarized, including gene engineering methods, stress-inducing conditions, chemical regulators, physical methods, and biomaterial stimulations. Through applying these strategies, increased yield of sEVs with required biological function can be obtained for disease treatment and tissue repair, such as bone regeneration, wound healing, nerve function recovery and cancer treatment, which could not only reduce the harvest cost of sEV but promote the practical applications of sEVs in clinic.

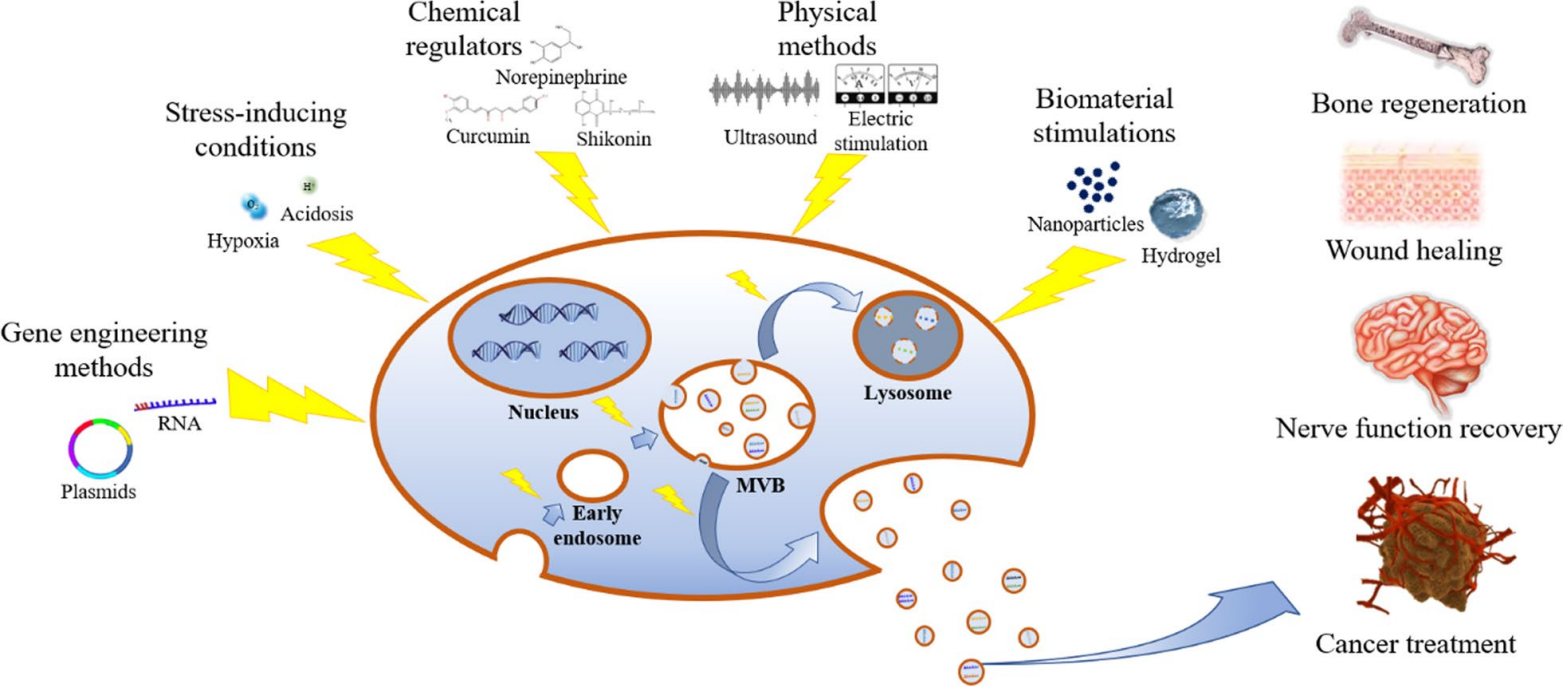

## Introduction

Small extracellular vesicles (sEVs) are a type of extracellular vesicles (EVs), with a size of 30–200 nm, comprised of a phospholipid bilayer membrane and sufficient bioactive substances, including proteins, mRNAs, miRNAs and lipids [1–3]. EVs are normally heterogeneous as they contain vesicles with different size, biological functions and some vesicles are released from different parts of cells or from different cellular status of a same type of cells [4]. EVs can be divided into exosomes, microvesicles, and apoptotic bodies, depending on their size, biological characteristics, biogenesis and release process [4]. Exosomes, which are typically 30–200 nm in size, are formed by the endosome system and display hallmarks of the intraluminal vesicles of endosomes [5]. However, heterogeneous populations of EVs from diverse biogenesis can be co-isolated into the exosome product when the size-based methods are used for exosome separation and purification [6, 7]. For example, microvesicles budding directly from the plasma membrane may also possess a diameter less than 200 nm and may be a part of exosome products since their size is in the similar range [8]. Thus, it is hard to remove non-endosome-origin vesicles from sEVs to get a pure exosome product. Therefore, it is more appropriate to use the term ‘sEVs’ to refer to EVs with a size of 30–200 nm in this review, which mainly include exosomes.

For the process and regulatory mechanisms of biogenesis and release of sEVs, most of the studies focused on exosomes, since they were the principal components of sEV populations isolated and purified by most of current methods [6, 7]. The process and regulatory mechanisms about biogenesis and release of exosomes have been reported in detail in many literatures [4, 9–11]. Briefly, exosomes are formed by the endosome system, which mainly go through three stages [9]. At first, the cell membrane forms endocytic vesicles, and multiple endocytic vesicles fuse together to form early endosomes. Then, early endosomes encapsulate specific intracellular ingredients and bud inward, forming multiple intraluminal vesicles (ILVs). In this process, early endosomes further transform into late endosomes, which are also called multivesicular bodies (MVBs). Finally, MVBs fuse with the cell membrane and release their contents. So that, ILVs are released outside the cell and become exosomes. According to the literatures, the biogenesis of exosomes can be divided into endosomal sorting complex required for transport (ESCRT)-dependent manner and ESCRT-independent manner [11]. ESCRT is mainly responsible for identifying ubiquitinated proteins (including misfolded proteins, activated cytokine receptors) in endosomal system and mediating the formation of intraluminal vesicles (ILVs) and multivesicular bodies (MVBs), while ESCRT-independent pathway is more complicated, mainly involving ceramide, tetraspanin, heat-shock protein (HSP) and lipids (Table 1). In addition, regulatory mechanisms of exosome release have also been studied, involving the Rab GTPases, soluble *N*-ethylmaleimide-sensitive fusion attachment protein receptors (SNARE), and some other regulatory molecules (Table 2) [10]. Understanding the process and regulatory mechanisms about biogenesis and release of exosomes is of great significance to develop strategies for regulating the production and biological function of exosomes.

Studies have demonstrated that sEVs can be secreted by almost all living mammalian cells and can be found in various body fluids [11]. Through delivering their cargos into recipient cells, sEVs can act as the media for cell–cell interactions and regulate cellular behaviors of surrounding or distant cells [8, 12]. Therefore, sEVs play an important role in physiological or pathological body activities. The biological function of sEVs that has been reported up to now include regulating immune response, promoting cell proliferation, promoting organ development, and controlling aging [4]. Since the sEVs are vital medium of intercellular paracrine communications, and sEV injection can perform similar biological function to cell transplantation in organism [13]. When compared with cell therapy, sEVs therapy can avoid the tumorigenicity, immune rejection and other potential risks [14, 15]. In addition, it is easy to store and transport sEVs, as well as modify their biological function. Therefore, it has great advantages for sEVs to replace cell transplantation in tissue regeneration and other disease treatment [16]. At present, extensive research related to sEV therapies have been carried out, including skin and cartilage damage repairing, neurological disease treatment, cardiovascular disease treatment, and cancer treatment [17].

However, facing up with the requirements of high dosage of sEVs in clinical application, especially in tissue regeneration, the production of sEVs by noncancerous cells is severely low [18]. In most studies, the production of sEVs is often less than 10^9^ particle per milliliter medium, while the effective dose of sEV therapy is usually more than 10^10^ particle per gram mouse. The practical applications of sEV therapies are severely restricted by the low production of sEVs. Although there are various methods for sEV separation and purification, such as ultracentrifugation, ultrafiltration, and size exclusion chromatography, these methods still have some disadvantages, including complex operation, expensive equipment, or poor separation efficiency [19–21]. The severely low sEV production further restricts the efficiency of separation and purification. Therefore, apart from improving the efficiency of sEV purification, increasing the capability of sEV production derived from donor cells is also of great significance.

In addition, for special applications, such as anti-inflammation, angiogenesis, bone regeneration, cartilage regeneration, and myocardial infarction repair, sEVs carrying different bioactive substances and performing specific biological function are required [17, 22]. Those sEVs with specific biological function can be obtained from specific type of donor cells [23]. For the donor cells of the same type, the inclusions of their sEVs are always different under different cellular states, along with significantly different potential in tissue repair. For one particular application, it is vital to strengthen the accumulation of required bioactive cargos in sEVs. Unfortunately, apart from the significant decline of sEV production, as cells aging, the component and biological function of sEVs are changing unmanageably [24].

Therefore, how to increase the sEV production from donor cell and enhance the required functions of sEVs is of great significance to promote the practical application of sEV therapies. There have been reviews that discuss the molecular mechanisms associated with regulation of sEV production and their specific cargo sorting, but the strategies for regulating sEV production and cargo sorting have not been discussed [9, 25]. This review summarizes current strategies and their possible applications to affect the behaviors of donor cells and subsequently regulate the production and biological function of their sEVs, including gene engineering methods, stress-inducing conditions, chemical regulators, physical methods, and biomaterial stimulations. According to the characteristics of each strategy and the actual application scenarios, researchers can choose appropriate strategies to obtain increased yield of sEVs carrying the desired therapeutic capability. These strategies will decrease the cost of sEV collection and promote the practical applications of sEV therapies in tissue repair/regeneration and disease treatments.

## Current strategies to regulate the production and biological function of sEVs

### Gene engineering technology

Gene engineering technology mainly needs to target at genes related to sEV biogenesis and release. Many reports have demonstrated that genes related to exosome biogenesis (Table 1) and release (Table 2) could be interfered by gene engineering technologies and subsequently exosome secretion from cells was affected. However, the relevance between these genes and exosome biogenesis and release could not be applied to different types of cells. For some genes, there has been no consensus about their roles in exosome biogenesis or release. In addition, mechanisms involved in exosome biogenesis and release are quite complex and heterogeneous, which makes it difficult to directly use gene engineering methods to regulate related genes of donor cells for obtaining high yield of exosomes with required function. In fact, most of other strategies regulates those genes and proteins related to exosome biogenesis and release in an indirect way.

**Fig. 1:**
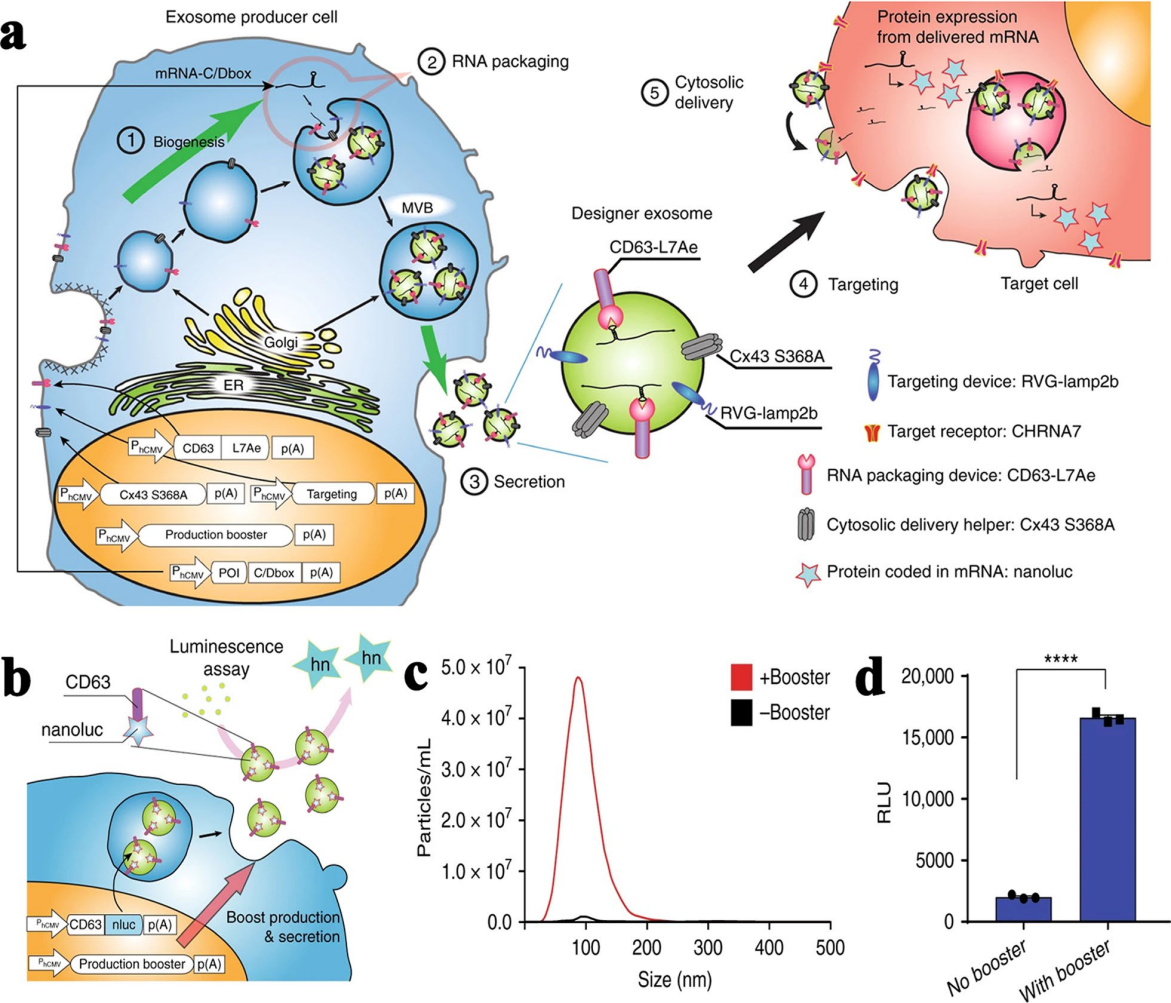
EXOtic devices to boost exosome production and deliver mRNA [29]. **a** Schematic illustration of the EXOtic devices. **b** Schematic illustration of luminescence assay for the quantification of exosome production. **c** Result of concentration and size distribution of exosomes. **d** Luminescence assay of CD63-nluc secreted into the supernatant reflected exosome concentration. All images are reproduced with permission from Kojima et al. [29]. Copyright © 2018 Author(s). All rights reserved

In addition to directly interfering with molecular mechanisms, specific nucleic acid can be transfected to donor cells and then be loaded into sEVs [12, 26–28]. So that, sEVs will better perform designed functions. The exosomal transfer into cells (EXOtic) device designed by Kojima et al. was confirmed to produce mass exosomes from donor cells [29]. Different plasmids were required to respectively boost exosome production, enhance RNA packaging, assist endocellular exosome secretion, and help exosomes to target recipient cells without being removed in vivo (Fig. 1a). In particular, the plasmid for exosome production boosting involved STEAP3 (related to exosome biogenesis), syndecan-4 (enhance inward budding of endosomal membranes to form ILVs), and a fragment of l-aspartate oxidase (accelerate the tricarboxylic acid cycle and increase cellular metabolism). Their result showed that combined expression of these three genes increased exosome production by 40-fold (Fig. 1c). The size distribution of generated exosomes hardly changed after these genes were expressed in cells (Fig. 1b, d). Furthermore, this exosome production booster was functional in multiple cell lines, including human mesenchymal stem cells (MSCs). With its generalizability, EXOtic can be a promising tool for efficient production of exosomes loaded with required therapeutic nucleic acids.

**Table 1:** Regulatory factors of exosome biogenesis

Regulatory factor	Correlation	Function
HRS [30, 31] STAM1 [32]	Positive correlated in various cells	HRS and STAM1 are members of ESCRT-0 complex which can recognize mono-ubiquitylated cargo proteins
TSG101 [33]	Positive correlated in various cells	TSG101 is a member of ESCRT-I complex and responsible for the budding of the endosomal membrane
CHMP4 [34]	Positive correlated in various cells	CHMP4 is member of ESCRT­III complex and perform fission of microdomains on the limiting membrane of MVEs, promoting vesicles separation
VPS4 [34]	Inhibition of VPS4b in HeLa cells increased exosome formation. Inhibition of both VPS4a and VPS4b in MCF-7 cells increased exosome formation, while inhibition one of VPS4a and VPS4b had no significant influence. Inhibition of both VPS4a and VPS4b in RPE1 cells inhibited exosome secretion	VASP4 plays an essential role in dissociation and recycling of the ESCRT complexes
ALIX [34]	Positive correlated in various cells	ALIX promotes endosome budding to form ILVs
nSMase2 [35]	Positive correlated in various cells	nSMase2 hydrolyzes sphingolipid to produce ceramide and promote MVBs budding inward
Cholesterol [36]	Positive correlated in oligodendroglia cell	They can sort related ligands into ILVs and sequestrate cytosolic proteins into ILVs
PLD2 [37, 38]	Positive correlated in MCF-7 cells	
CD63 [39]	Positive correlated in MNT-1 melanoma cells	
CD9 [40], CD82 [40]	Positive correlated in HEK293 cells	
CD81 [41]	Positive correlated in human primary lymphoblasts	
TSPAN8 [42]	Positive correlated in rat pancreatic adenocarcinoma cells	
HSP70 [43, 44]	Positive correlated in various cells

**Table 2:** Regulatory factors of exosome release

Regulatory factor	Correlation	Function
Rab2b [45] Rab5a [45] Rab9a [45]	Positive correlated in HeLa cells	Rab GTPases regulate different stages of vesicle transport, including budding, movement and connection, thus promoting the fusion of MVBs and plasma membrane
Rab11 [46, 47] Rab27a [45, 48–50] Rab27b [45, 49, 50] Rab31 [51] Rab35 [52, 53]	Positive correlated in various cells	
Rab7 [34]	Positive correlated in MCF-7 cells	
SNAP23 [54] VAMP7 [55] VAMP8 [56]	Positive correlated in various cells	They participate in Ca^2+^-regulated fusion of organelles membrane and plasma membrane
Ykt6 [57]	Positive correlated in HEK293 cells	Ykt6 promotes the secretion of exosomes containing Wnt3a
STX5 [58]	Positive correlated in 4T1 cells	They participate in the fusion of MVBs and plasma membrane
STX1a [59]	Positive correlated in S2 cells	
DGKα [60]	Negative correlated in T lymphoblasts	DGKα inhibits the formation of MVBs

### Stress-inducing conditions

Stress-inducing conditions, such as hypoxia, starvation, acidosis and inflammation, can cause a variety of molecular changes and cellular responses. The initial processes involved in cellular stress responses tend to minimize acute damage and promote cell survival against unfavorable environmental conditions [61]. Excessive intensity and duration of stress-inducing conditions will elicit programmed cell death that eliminates damaged cells [61]. Undoubtedly, stress-inducing conditions affect sEV secretion in various types of cells. Rapid proliferation of cancer cells usually leads to hypoxia, acidic pH, insufficient blood supply and lack of nutrition in tumor microenvironment [62]. Under stress-inducing conditions, cancer cells communicate more frequently with other cancer cells and surrounding non-cancer cells to promote cancer cell survival, so sEVs production is enhanced, as a media of these intercellular communications [63]. For example, after being exposed to hypoxic microenvironment, breast cancer secreted more sEVs with packaged hypoxia-related miRNAs, and this process occurred in a hypoxia-inducible factor 1α dependent manner [64]. It has been reported that the sEVs secreted under hypoxic condition also contained more STAT3 and FAS, which could be transferred to other cancer cells to promote tumor growth and metastasis [63]. In addition, sEVs from glioblastoma cells cultured in hypoxic microenvironment could induce angiogenesis and tumor growth, possibly by exchanging hypoxia related RNAs and proteins [65]. Hypoxia in tumors is usually related to increased glycolysis and accumulation of lactic acid, which results in an acidic microenvironment. Thus, the intracellular pH also affects sEVs secretion. Acidic pH (pH = 6.0) increased sEV secretion and uptake in melanoma cells, and alkaline pH (pH = 11) decreased the secretion of sEV and the synthesis of sEV-related proteins and RNA in HEK-293 cells [66, 67]. Although acidic pH increased the release of sEVs, storage of sEVs in acid solution (pH = 4.0) caused exosomal protein degradation. This acidic condition (pH = 4.0) was beyond the typical pH range of the tumor microenvironment (pH 6.5–6.9), but long-term stability of sEVs might be impaired in acidic environment, thereby resulting in the destruction of sEV physiological functions [68]. Gong et al. also found sEVs derived from human gastric cancer cells cultured in acidic condition (pH = 4.0) had an increased uptake efficiency, resulting from a glycerolipid self-aggregation-based mechanism for the enhanced homologous uptake [69]. In addition, Fan et al. reported sEVs produced under glutamine depletion condition could promote tumor angiogenesis, tumor cell proliferation and tumor metastasis [70]. Glutamine depletion reduced growth regulatory Akt/mTORC1 signaling and increased release of Rab11 + sEVs from cancer cells [70]. This study suggested that glutamine supplements might reverse the inhibition of Akt/mTORC1 signaling in the tumor environment and reduce the release of sEVs related to tumor growth and metastasis, which might be beneficial to cancer treatment.

**Fig. 2:**
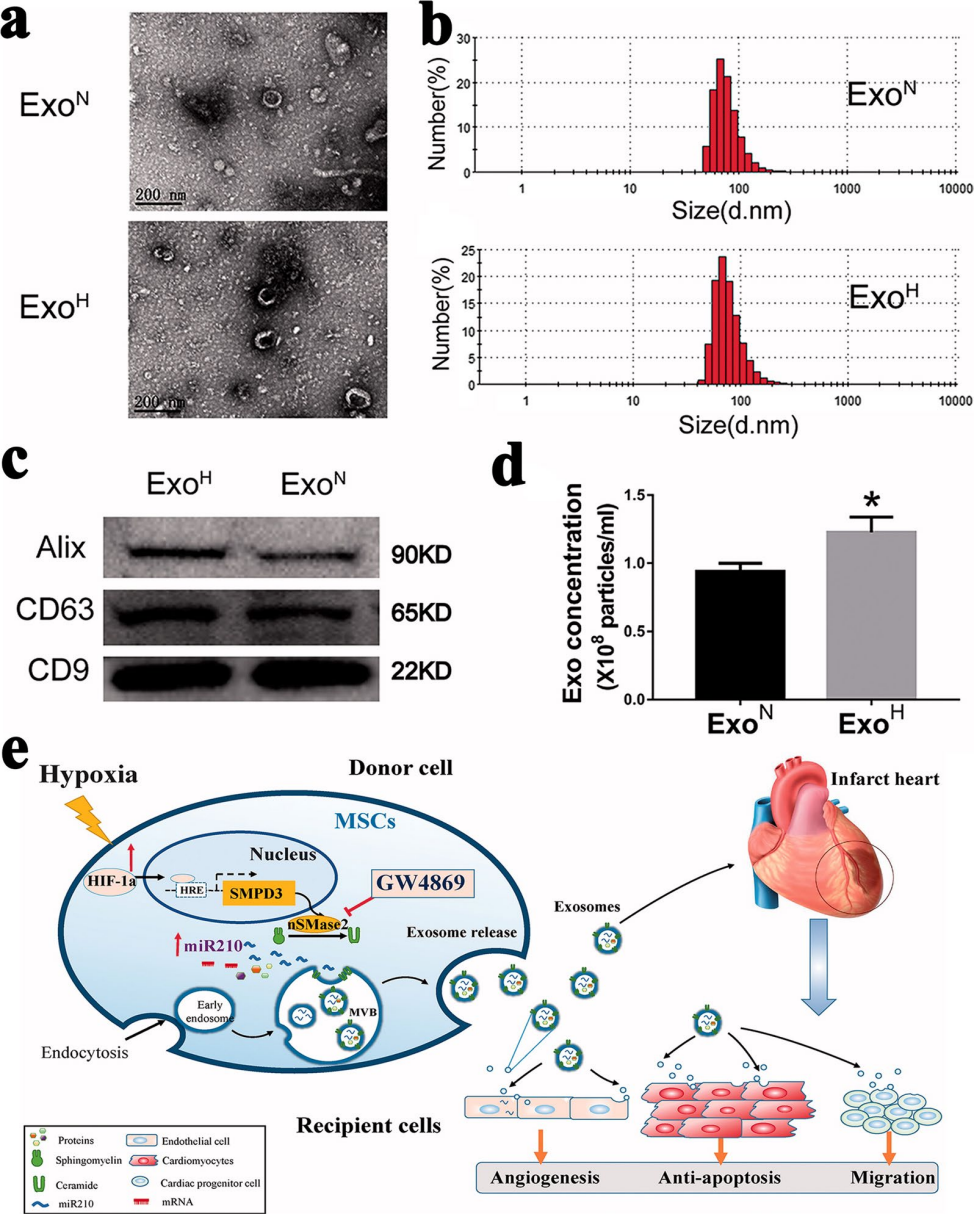
Enhanced production and cardiac-repair capability of exosomes derived from hypoxic-conditioned MSCs [78]. **a** Cup-shaped morphology of purified exosomes assessed by transmission electronic microscope. **b** Size distribution of exosomes. **c** Exosomal markers assessed by Western blotting. **d** Number of exosome particles assessed by nanoparticle tracking analysis (NTA). **e** Schematic representation of the effects and mechanisms of hypoxic-MSCs derived exosomes for cardiac repair after myocardial infarction. All images are reproduced with permission from Zhu et al. [78]. Copyright © 2018 Taylor & Francis. All rights reserved

For noncancerous cells, studies on stress-inducing conditions changing sEV secretion mainly focused on hypoxia and inflammation condition. After pathogen infection or exposure to inflammatory environment, most types of cells produced proinflammatory sEVs and caused subsequent immune responses [71–73]. Wang et al. found that the sEVs derived from M1-Polarized macrophages enhanced the anti-tumor effects of paclitaxel by activating macrophages-mediated inflammation [71]. Nevertheless, the sEVs derived from MSCs in inflammatory or infected environment always show an anti-inflammatory and immunosuppressive activity [74–76]. Ti et al. found sEVs derived from lipopolysaccharide preconditioned MSC had a better ability to upregulate the expression of anti-inflammatory cytokines in macrophages and promotion of M2 macrophage activation [74]. Huang et al. reported that the miRNAs in MSC-derived sEVs were changed by inflammatory cytokines, which were related to immunosuppressive functions and angiogenesis [76]. Meanwhile, they also found that vascular cell adhesion molecule-1 stimulation resulted in a 1.5-fold increase of sEV production from MSCs, whereas interleukin 6 stimulation reduced sEV production to 40% [76]. Hypoxia is another stress-inducing condition and the sEVs derived from hypoxia-conditioned donor cells have been proved to be beneficial to ischemic cardio-cerebrovascular disease and angiogenesis in tissues [77–79]. For example, sEVs derived from rat cardiomyocyte progenitor cells (CPCs) under hypoxic microenvironment showed significantly stronger ability of enhancing tube formation of endothelial cells (ECs) and reducing profibrotic gene expression in fibroblasts stimulated by transforming growth factor β (TGF-β) [77]. Microarray analysis of sEVs secreted by hypoxia-conditioned CPCs indicated that at least six miRNAs related to cardiac function were upregulated. Similarly, when bone marrow-derived MSCs were cultured under hypoxic condition, the cup-shaped morphology, size distribution and exosomal markers of exosomes secreted by cells cultured under hypoxic condition (Exo ^H^) were similar to those of exosomes obtained from cells cultured under normoxic condition (Exo ^N^) (Fig. 2a–c). However, the number of Exo ^H^ was 1.3-fold higher than that of Exo ^N^ (Fig. 2d) [78]. After being injected into mouse infarcted heart, Exo ^H^ showed better cardiac function recovery as compared to Exo ^N^. Further study on the mechanism found that hypoxia pre-treatment of MSCs resulted in an increase expression of (hypoxia-inducible factor)-1α and nSMase2 in cells and an upregulation of miR-210 level in exosomes, which were respectively associated with exosome secretion and cardiac function (Fig. 2e). Besides, Zhu et al. found that miR-125b-5p was enriched in hypoxia-conditioned MSCs-derived sEVs, which could inhibit the expression of cardiomyocyte proapoptotic gene p53 and BAK1 [79]. Therefore, these sEVs promoted cardiomyocytes survival, effectively reduced the area of myocardial infarction, and finally facilitated cardiac repair.

### Chemical regulators

Chemical regulators have also been applied to regulate the production and biological function of sEVs. Datta et al. used quantitative high-throughput to screen the inhibitors and activators of sEV secretion in aggressive prostate cancer CD63-GFP-expressing C4-2B cells among 4580 compounds from the LOPAC library (1280 pharmacologically active compounds) and the NPC library (3300 compounds approved for clinical use) [80]. Their study identified several compounds (norepinephrine, *N*-methyl dopamine, mephenesin, etc.) that could enhance the production of C4-2B prostate cancer cell-derived sEVs. On this basis, Wang et al. further studied the effects of five compounds on sEV secretion of MSCs [81]. Interestingly, fenoterol, *N*-methyl dopamine, and mephenesin failed to increase the secretion of MSC-derived sEVs at low doses (10 μM), and only succeeded at high doses (50 μM fenoterol, 50 μM *N*-methyl dopamine, and 100 μM mephenesin). However, 10 µM fenoterol, *N*-methyl dopamine, and mephenesin induced a 3.6-fold, 4.4-fold, and 3.4-fold increase in sEV secretion from C4-2B prostate cancer cells, respectively. Similarly, when 10 µM norepinephrine and forskolin were tested in C4-2B prostate cancer cells, the sEV secretion from these cells was enhanced by 4.6-fold and 5.7-fold, respectively, while 10 µM norepinephrine and forskolin both merely increased MSC-derived sEV secretion by twofold. When testing the effects of compound combination on MSCs sEV secretion, the co-administration of norepinephrine and *N*-methyl dopamine had the most significant enhancement effect (threefold) on sEV production. This enhancement in sEV secretion is not due to an increase in cell number after chemical modulators treatment but could be the result of enhanced metabolic activities [81]. Besides the regulatory effect on sEV production, several chemical regulators capable to modulate the biological function of sEVs have also been identified. Hu et al. found sEVs derived from pioglitazone pretreated MSCs better recovered the cell viability and proliferation of human umbilical vein ECs from high glucose injury and promoted diabetic wound healing through enhancing angiogenesis [82]. Ibrahim et al. reported 6-bromoindirubin-3′-oxime could increase β-catenin levels and activate Wnt signalling in cardiosphere-derived cells, and then lead to upregulation of miR-92a in sEVs. Those modulated sEVs showed improved contractility and attenuation of fibrosis in heart [83].

In addition, many chemical components extracted from plant and animal products have been demonstrated to have a unique regulatory effect on the production and function of sEVs derived from specific cells, and then improve the therapeutic effect of sEVs on specific diseases (Table 3). These results strongly suggested that sEV secretion was a cell-specific process, and chemical regulators might not have the same effects on all types of cells. Therefore, chemical modulator is a convenient tool to increase the production of sEVs. Nevertheless, the mechanism behind these newly discovered chemical regulators is still not fully understood, and their potential influence on sEV contents and biological function is also unclear. Further investigation on chemical regulators is needed before their practical use in sEV production. Especially for developing anti-cancer drugs, elucidating the mechanisms through which the chemical regulators modify the sEV production is critical for designing novel inhibitors that possess the capacity to specifically target the sEV secretion of cancer cells (Table 4).

**Table 3:** Regulatory effect on sEVs by compounds extracted from plant drugs

Compound	Donor cells	Recipient cells	Disease	Regulatory effect
Curcumin [84]	H1299 cells	BEAS-2B cells	Lung cancer	Curcumin exerts its anti-cancer function by downregulating DNMT1, thereby upregulating exosomal TCF21
Curcumin [85]	Mouse brain ECs	Mouse brain ECs	Blood–brain barrier disruption	The sEVs derived from curcumin-treated MBECs alleviated oxidative stress, tight junctions (ZO-1, claudin-5, occludin), adherent junction (VE-cadherin) proteins and EC layer permeability induced during EC damage due to hyperhomocysteinemia
Shikonin [86]	Mouse preadipocytes	MCF10DCIS cells	Breast cancer	Shikonin-treated preadipocytes secreted sEVs with high levels of miR-140, which can inhibit nearby ductal carcinoma in situ cells. through targeting SOX9 signaling
Shikonin [87]	MCF-7 cells	MCF-7 cells	Breast cancer	Shikonin inhibits the proliferation of MCF-7 cells through inhibiting sEV release and reducing tumor-derived exosomal miR-128
Berberine [88]	Glomerular mesangial cells	Podocytes	Diabetic nephropathy	Berberine significantly ameliorated the injury of podocytes induced by ((high glucose)-induced glomerular mesangial cell)-derived sEVs, likely through downregulating TGF-β1 content in sEVs
Halofuginone [89]	MCF-7 cells	MCF-7 cells	Breast cancer	Inhibition of sEV production by halofuginone reduces exosomal miR‐31, which targets the histone deacetylase 2/cell cycle signaling axis and further inhibits MCF‐7 cell growth
β-Elemene [90]	MCF-7 cells*	MCF-7 cells*	Breast cancer	β-Elemene altered the expression of some multidrug resistance related miRs, including PTEN and Pgp in cells and their sEVs, reversing drug resistance
Docosahexaenoic acid [91]	MCF-7, MDA-MB-231, ZR751 and BT20 cells	EA.hy926 ECs	Breast cancer	Docosahexaenoic acid enhanced the sEV secretion of breast cancer cells and increased exosomal miRNAs related to anti-cancer and/or anti-angiogenic activity (let-7a, miR-23b, miR-27a/b, miR-21, let-7, and miR-320b)
Tetramethyl-pyrazine [92]	Cardiac MSCs	Cardiac MSCs	Ischemic heart disease	Tetramethylpyrazine treatment increased sEVs release from Cardiac-MSCs through upregulating the Rab27a, SYTL4 and Rab27b proteins

*Donor cells and recipient cells are both adriacin-resistant MCF-7 cells and docetaxel -resistant MCF-7 cells

### Physical methods

Physical methods (ionizing radiation, electric field, ultrasound, external force, etc.) also exert great influences on sEV secretion. For example, it was found that the sEV secretion by cancer cells increased significantly after radiotherapy [93]. Studies have demonstrated that exposure to ionizing radiation caused DNA damage in cells and the p53 transcription factor activated by DNA damage stimulated the expression of transmembrane protein tumor suppressor activator 6, which activated additional pathways for sEV biogenesis and release [94]. Ionizing radiation on cells could also cause changes on exosomal compositions. It has been found that B7-H3 (CD276), EIFs, Rabs, CTGF mRNA increased in sEVs derived from different kinds of cancer cells affected by different dose and duration of ionizing radiation [94]. In addition, the effects of ionizing radiation can cause different biological function changes on sEVs. On one hand, the effects of ionizing radiation on sEV biological function could promote the migration of recipient cancer cells [93]. On the other hand, the communication between irradiated and unirradiated surrounding cancer cells mediated by sEVs gave rise to bystander effect, causing the unirradiated recipient cancer cells to be injured, like those irradiated cancer cells [93]. If further study can uncover the mechanism behind the sEV-mediated injury of unirradiated surrounding cancer cells, engineered sEVs derived from irradiated cancer cells in vitro have great application potential for cancer therapy.

**Fig. 3:**
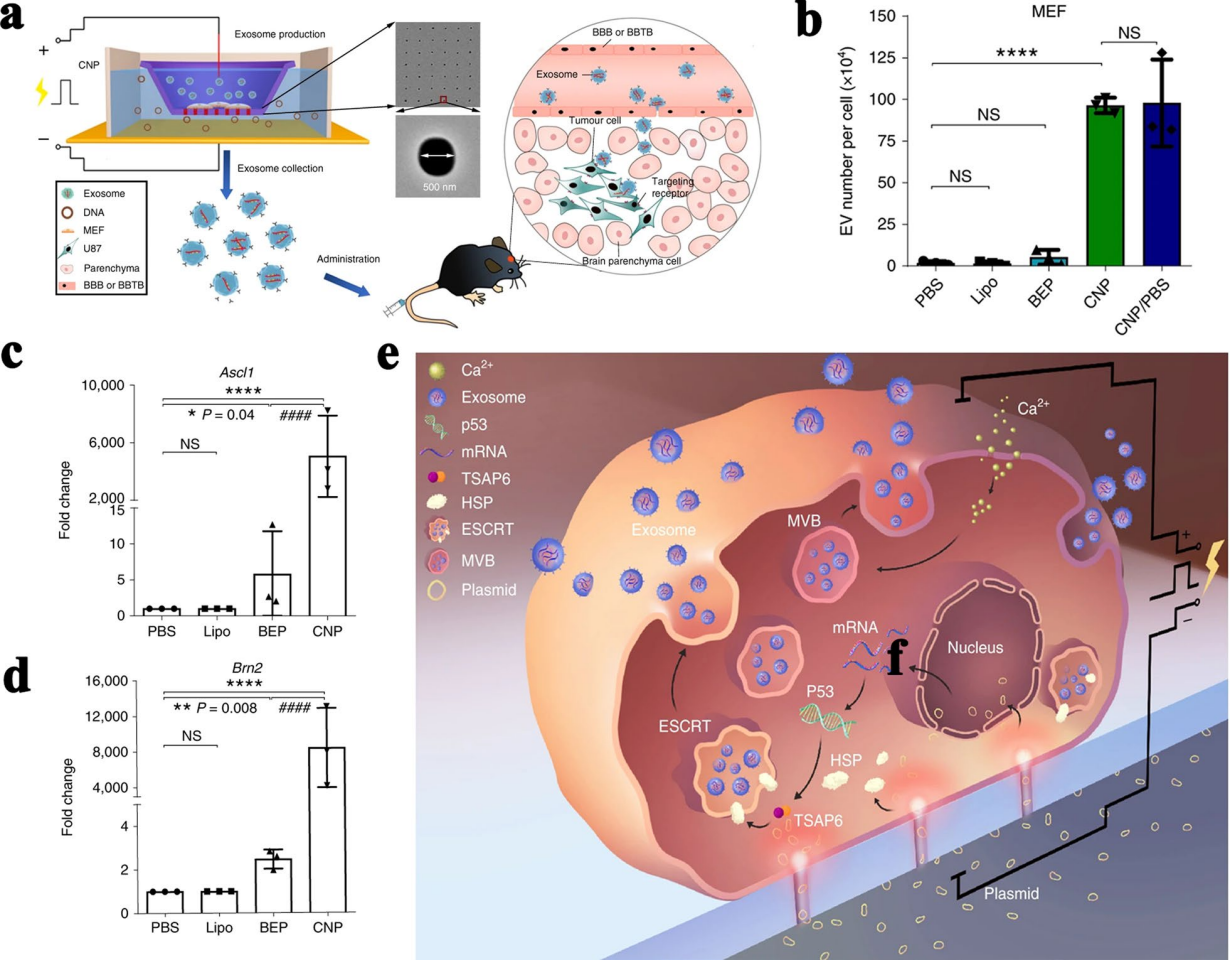
Large-scale generation of functional mRNA-encapsulating exosomes via CNP [97]. **a** Schematic representation of CNP-generated exosomes for targeted nucleic acid delivery. **b** Number of EVs. **c**, **d** Fold change of Ascl1and Brn2 mRNA in EVs from CNP-transfected MEFs. **e** Schematic of a proposed mechanism for CNP triggering of exosome release in CNP-transfected cells. All images are reproduced with permission from Yang et al. [97]. Copyright © 2019 Author(s). All rights reserved

As a common method for cell fusion and nucleic acid transfection, electric stimulation also had obvious influence on sEV secretion. Fukuta et al. demonstrated that low level electricity (0.3–0.5 mA/cm^2^) could induce the activation of intracellular signaling including Rho GTPase and subsequent endocytosis of extraneous molecules, which resulted in the promotion of sEV secretion [95, 96]. After 60-min electric treatment (0.34 mA/cm^2^) on murine melanoma and fibroblast cells, the particle number of sEVs isolated by ultracentrifuge increased by 1.26-fold and 1.7-fold, respectively. Lee et al. developed a cellular nanoporation (CNP) biochip, which transfected various donor cells with plasmid DNAs and stimulated donor cells with a focal and transient electrical stimulation to produce exosomes containing nucleotide sequences of interest (Fig. 3a) [97]. Compared with lipofectin transfection and bulk electroporation, CNP produced up to 50-fold more exosomes and caused a more than 10^3^-fold increase in mRNA transcripts (Fig. 3b–d) derived from mouse embryonic fibroblasts. Meanwhile, exosomes produced by CNP did not change ubiquitous exosomal markers and internalization capacity. The work principle of this biochip can be summarized as: focal cell-membrane injuries and local heating from CNP resulted in upregulation of HSPs and elevated intracellular Ca^2+^ concentration, leading to the formation of more ILVs (Fig. 3e). These ILVs were released into extracellular space as exosomes which could be induced to contain therapeutic RNAs after plasmid DNA delivery. Although the precise molecular and cellular mechanisms involved in electric stimulation-increased sEV production is still under investigation, electric stimulation is a powerful tool to produce large yield of sEV carrying therapy nucleic acids. To some extent, CNP can serve as a sEV-mediated gene transfection and can efficiently transfect nucleic acids with known function into target cells of specific tissue/organ. However, CNP caused intense stimulation on donor cells to transitorily release large amounts of sEVs. It might be difficult for CNP to maintain the steady production of sEVs with customized biological function.

Mechanical stimulation has a great influence on cellular behaviors, especially on the proliferation and differentiation of stem cells [98, 99]. Mechanical stimulation can activate stretch-activated channels and integrins on the plasma membrane, leading to cytoskeletal remodeling and changing gene expression of cells [98]. The effects of mechanical stimulation on sEV secretion were also reported. Wang et al. demonstrated that cyclic stretch force strongly induced periodontal ligament cells to secrete sEVs (more than 20-fold increase sEV production calculated by the mass of exosomal protein), and that sEVs better inhibited IL-1β production in lipopolysaccharide/nigericin-stimulated human macrophages [100]. Guo et al. seeded dental pulp stem cells and MSCs in 3D FibraCel scaffolds to apply flow stimulation (0.5 mL/min) on cells, and sEV production was 24-fold and 3.4-fold higher than the 3D static counterparts, respectively [101]. They also apply cyclic stretching on skeletal muscle cells (SkMCs) seeded into 3D-printed PDMS elastic scaffolds, and SkMCs produced 11-fold higher sEV yield than the unstretched SkMCs [101]. This mechanical-stimulated-enhancement of sEV production was mediated by yes-associated protein mechanosensitivity.

**Fig. 4:**
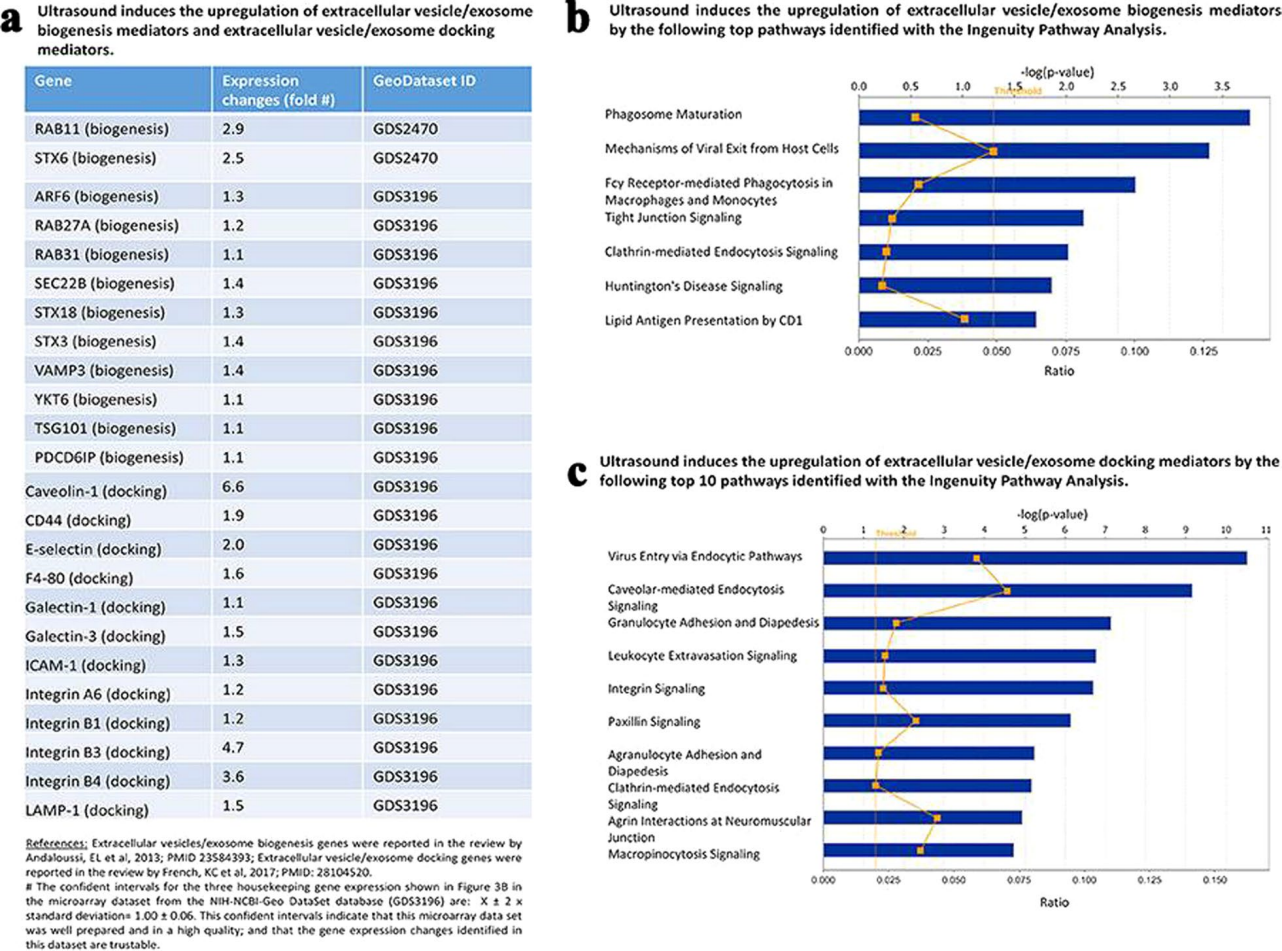
LIUS therapy increases markers of sEV biogenesis and docking [104]. **a** List of sEV biogenesis and docking genes that were upregulated with LIUS therapy. **b** Signaling pathways that are affected by the sEV biogenesis genes that are upregulated with LIUS treatment. **c** Major signaling pathways that are regulated by the sEV docking genes that had increased expression with LIUS therapy. All images are reproduced with permission from Yang et al. [104]. Copyright © 2017 Author(s). All rights reserved

Ultrasound has been widely used in medical fields, including imaging, lithotripsy, drug loading and delivery [102, 103]. Ultrasound have complicated physical effects on cells and tissue, such as heat, pressure, shockwaves, micro jets, and shear stress [104]. These physical effects can transiently puncture plasma membrane and cause concomitant perturbation of actin cytoskeleton, which profoundly affect cellular behaviors and gene expression [105]. Recently, numerous studies verified the ability of ultrasound in promoting anti-inflammatory and tissue repair/regeneration, expanding the applications of ultrasound in medical field [105, 106]. Anti-inflammatory effects induced by low-intensity ultrasound (LIUS) is mainly due to LIUS promotes the sEV secretion of several immunosuppressive cells, like myeloid-derived suppressor cells, mesenchymal stem cells and immunosuppressive dendritic cells. Yang et al. found LIUS significantly induced the expression of several sEV biogenesis mediators, of which Rab11 and STX6 were upregulated to 2.9-fold and 2.5-fold, respectively (Fig. 4a) [104]. Ingenuity Pathway Analysis on sEV biogenesis genes and docking genes upregulated by LIUS exposure was conducted to determine the major signaling pathways affected by those upregulated genes (Fig. 4b, c). Li et al. found sEVs derived from low-intensity pulsed ultrasound (LIPUS)-treated Bone marrow dendritic cells (BMDCs) impeded TNFα-induced activation of the NF-κB signaling pathway, thus better inhibiting TNFα-induced endothelial inflammation [107]. The possible mechanism was that sEVs derived from LIUS-treated BMDCs were rich in miR-16 and miR-21. Maeshige et al. used LIPUS of 3.0 W/cm^2^ to stimulate C2C12 myotubes and increased the number of sEVs by twofold at 6 h. The potential mechanisms may be that the micro jets generated by ultrasound caused pore formation through the plasma membrane. In addition, on several types of cancer cell lines, ultrasound irradiation was also found to promote the production of sEVs, such as SPC-A1, A2780, GL261, and U87-MG cells [108–111]. Although ultrasound stimulation was found to promote sEV yields of various cell types, the poor reproducibility of these research results hindered the practical application of ultrasound. It mainly resulted from the uncertainty in the multiple ultrasound field parameters experienced by sonicated donor cells [112]. Therefore, it is necessary to develop more stable and controllable equipment for ultrasound output. Moreover, extensive investigations are needed to find the proper ultrasonic parameters that adapt to donor cells with different types, and even further to induce the accumulation of specific constituent in sEVs to achieve customized biological function.

### Biomaterial stimulation

Biomaterials refer to native or artificial materials that can diagnose, treat, replace, repair, or induce regeneration of tissues and organs [113]. The bioactive signals of biomaterials (including chemical cues, structural cues, and mechanical cues) can affect behaviors of cells, actively regulate gene expression, and then affect metabolism of cells [114–116]. Once cell behaviors are regulated by biomaterials, sEV secretion will be regulated accordingly. In addition, in damaged tissues and organs, the paracrine effects between cells can promote cell recovery and proliferation, which is of great significance for tissue repair/regeneration [117]. Numerous reports have demonstrated that different bioactive signals of biomaterials have obvious regulatory effects on paracrine effects [114]. Since the sEVs are important media of paracrine effects, it can be reasoned that biomaterials should have regulatory effects on sEV secretion. Utilizing the regulatory effects of biomaterials on cell behaviors to customize sEVs with required functions has promising application prospects [118].

Nanoparticles are an important category of biomaterials. The balance between degradative and secretory capacity of MVBs significantly affects cell exosome production [9]. After phagocytized by donor cells, nanoparticles can enter lysosomes and prevent the degradation of MVBs, thus promoting the release of ILVs. Park et al. developed positively charged nanoparticles based on iron oxide and poly lactic-co-(glycolic acid) [119]. After internalized by MSCs and then transported to lysosome, these nanoparticles could upregulate the expression of Rab7 and enhance the production of sEVs. Furthermore, several specific antioxidants or tissue regeneration factors were enriched in sEVs derived from MSCs exposed to nanoparticles. Zhu et al. found that respiratory exposure to magnetic iron oxide nanoparticles (MIONs) increased the number of sEVs secreted by mouse antigen-presenting cells (APCs) in alveoli [120]. Meanwhile, sEVs produced by APCs exposed to MIONs could induce series of immune responses, including promotion of immature dendritic cells (DCs) maturation and DC1 subtype differentiation, macrophage activation and M1 subtype differentiation of macrophages. Moreover, more than one strategy could be combined to enhance the exosome production and improve biological function of exosomes. Wu et al. combined 50 µg/mL Fe3O4 magnetic nanoparticles and 100 mT static magnetic feld stimulation on bone MSCs [121]. Compared with exosomes derived from normal bone MSCs, the exosomes derived from these modulated bone MSCs was increased produced and these exosomes better improved osteogenesis and angiogenesis [121]. They also found the improvement resulted from the rich miR-1260a in exosomes derived from these modulated bone MSCs. Shyong et al. demonstrated that treatment with Calcium phosphate (CaP) particles could increase over twofold sEV production from macrophage-like RAW264.7 cells and monocyte-like THP-1 cells [122]. Moreover, Ca^2+^ contents in sEVs derived from CaP particle-treated cells were similar to that in sEVs from untreated control cells. CaP particles were easily internalized into cells and dissolved in acidic lysosomes. High concentration of Ca^2+^ in the cytosol promoted the fusion of MVBs with plasma membrane [122].

**Fig. 5:**
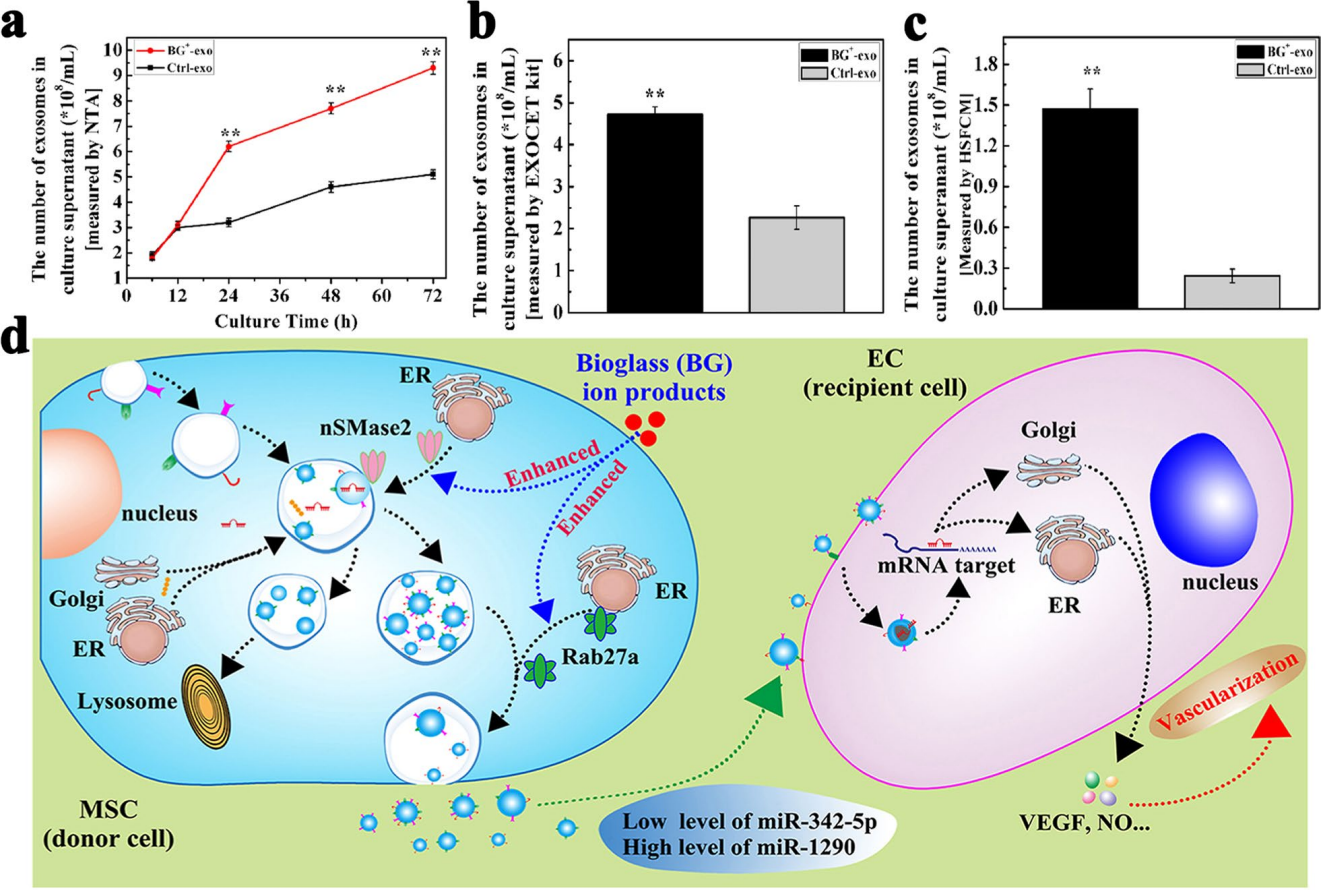
Bioglass enhances the production of exosomes and improves their capability of promoting vascularization [125]. **a** The number of exosomes after MSCs were cultured with BG ion products for 6–72 h. **b**, **c** The number of exosomes after MSCs stimulated by BG ion products for 48 h. **d** Proposed underlying mechanisms of BG chemical cues (ion products) in enhancing the production and modifying the function of MSCs-derived exosomes. All images are reproduced with permission from Wu et al. [125]. Copyright © 2020 Author(s). All rights reserved

The influences of different bioactive signals from biomaterials on sEV secretion were confirmed. Zhang et al. prepared micro/nanonet-textured hierarchical titanium topography through the combination of selective laser melting and alkali-heat treatment techniques and they confirmed that the special titanium topography (structural cues) enhanced sEV biogenesis and extracellular secretion by upregulating Rab27b and SMPD3 gene expression [123]. Wu et al. cultured Huh7 cells on low stiffness (500 Pa) and high stiffness (10 kPa) acrylamide hydrogel (mechanical cues), respectively, and found that high matrix stiffness activated intracellular Akt, Rabin8 and Rab8, thereby promoting sEV secretion [124]. It was also found that sEVs produced by cells cultured on high stiffness matrix could promote tumor growth [124]. Our previous study also showed that stimulating human derived MSCs with bioglass (BG) ion products (chemical cues) could upregulate the expression of nSMase2 and Rab27a, activating related signaling pathways of exosome biogenesis and release and significantly enhancing exosome production (Fig. 5) [125]. BG ion products brought a more than double increase of MSC-derived exosome production (Fig. 5a–c). In addition, compared to the exosomes derived from unstimulated MSCs, exosomes derived from MSCs stimulated by BG ion products showed improved capability to promote ECs vascularization. According to the result of miRNA array analysis, the downregulation of miR-342-5p and the upregulation of miR-1290 in exosomes were related to VEGF signaling pathway in ECs and affect the vascularization of ECs (Fig. 5d).

## Applications of modulated sEVs in disease treatments

### Modulated sEVs for cancer treatments

The sEVs play an important role in occurrence and development of cancers. sEVs can carry carcinogenic factors and transport them to other cancer cells, surrounding noncancerous cells and immune cells [6]. The communication between cancer cells and other cells mediated by sEVs promotes cancer cells proliferation and invasion, and lead to immunosuppression, helping cancer cells to evade the removal of immune system. Inhibition of cancer cell sEV secretion can inhibit tumor progression, reduce cancer metastasis, and relieve tumor immunosuppression, which is of positive significance for cancer treatment [87, 126]. Several natural compounds extracted from plant and animal product also have regulatory effect on sEVs which can play an anti-cancer role later, such as shikonin (Table 3) [87].

**Fig. 6:**
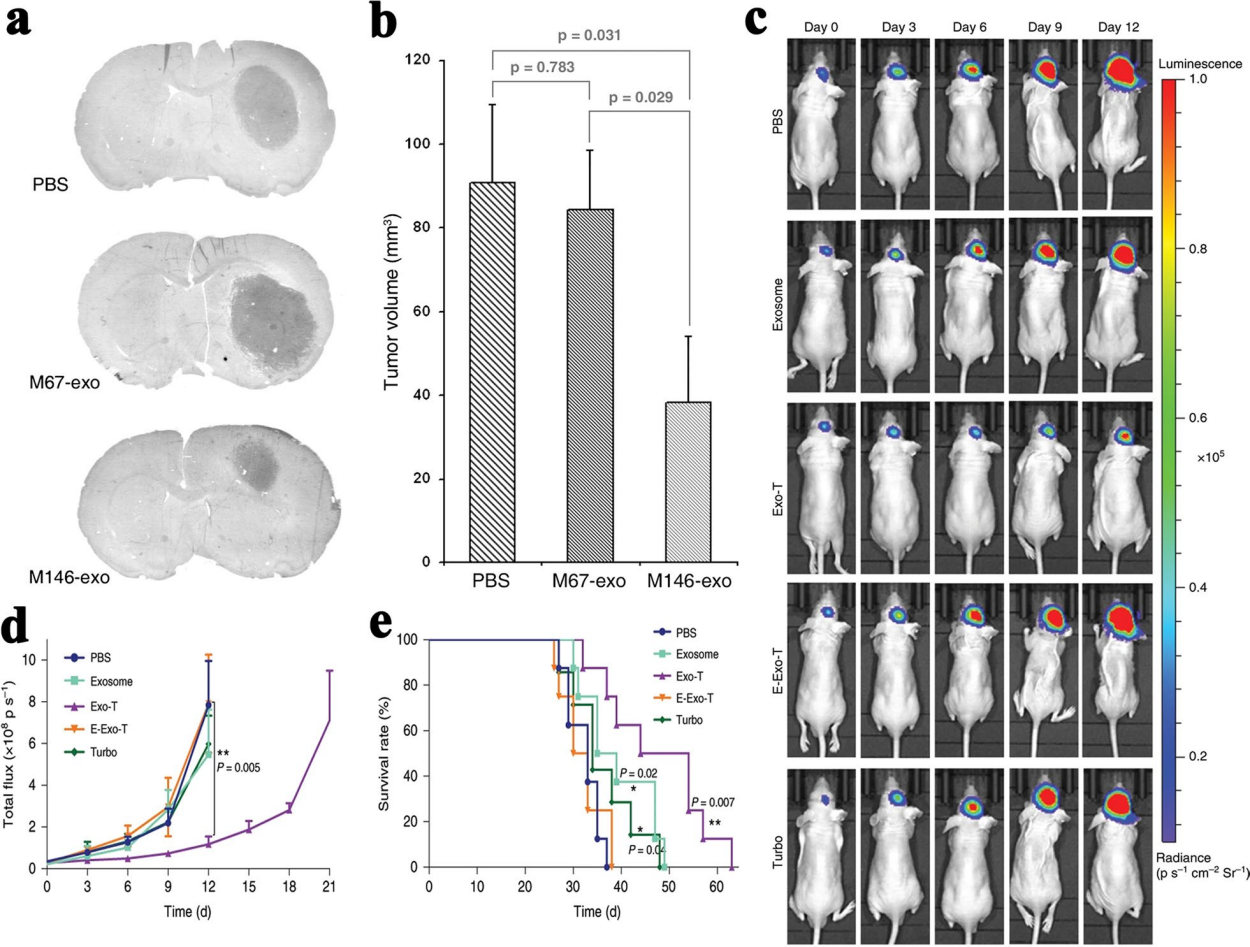
Modulated sEVs for cancer treatment [28, 97]. **a**, **b** Intra-tumor injection of exosomes derived from miR-146-expressing MSCs reduced 9L glioma growth in rat brain. All images are reproduced with permission from Katakowski et al. [28]. Copyright © 2013 Elsevier Ireland Ltd. All rights reserved. **c**–**e** mRNA-containing exosomes produced by CNP enhanced inhibition of tumor growth and increased survival in orthotopic PTEN-deficient glioma mouse models. Exosome: normal, Exo-T: treated by CNP and contain PTEN mRNA, E-Exo-T: treated by CNP and empty with PTEN mRNA, Turbo: TurboFect nanoparticles. All images are reproduced with permission from Yang et al. [97]. Copyright © 2019 Author(s). All rights reserved

In addition to promoting the progress of cancers, sEVs can also serve as means of cancer treatment. sEVs naturally exist in all body fluids and have fine biocompatible and biodegradability [127]. Compared to other drug carriers, sEVs have lower toxicity and immunogenicity. Meanwhile, sEV therapy may be a new method for treating brain malignancies with their ability of crossing the blood–brain barrier [15]. For efficient drug delivery, drugs can firstly be ingested into cell. Then, these drugs will be load into sEVs with the formation process of sEVs [128]. Ingesting specific drug can sometimes stimulate donor cells to produce other therapeutic substances which will be packed into sEVs [84, 86]. Nucleic acid can also be transfected into cells through gene engineering or physical methods, and then therapeutic RNA and proteins can be loaded into sEVs [12]. The miR-146b expression plasmid was transfected into the MSCs through electroporation, and then miR-146b was loaded into MSCs-derived exosomes (7-to-eightfold higher) [28]. Intra-tumor injection of exosomes derived from miR-146-expressing MSCs significantly reduced glioma xenograft growth in a rat model of primary brain tumor (Fig. 6a, b) [28]. Villarroya-Beltri et al. found that short sequence motifs over-represented in miRNAs (EXOmotifs) could guide their loading into sEVs [129]. The directed mutagenesis of EXOmotifs enabled the modulation of miRNA cargo in these vesicles. Heterogeneous nuclear ribonucleoprotein A2B1 bound a specific subset of miRNAs through recognizing EXOmotifs and led their loading into sEVs [129]. Therefore, miRNAs transfected into donor cells could be loaded into sEVs more efficiently with EXOmotifs. Lee et al. applied cellular nanoporation to make large-scale generation of functional mRNA-encapsulating exosomes [97]. In orthotopic phosphatase and tensin homologue (PTEN)-deficient glioma mouse models, mRNA-containing exosomes restored PTEN expression, recovered tumor-suppressor function, enhanced inhibition of tumor growth and increased survival rate of mice (Fig. 6c–e).

### Modulated sEVs for tissue repair and regeneration

**Fig. 7:**
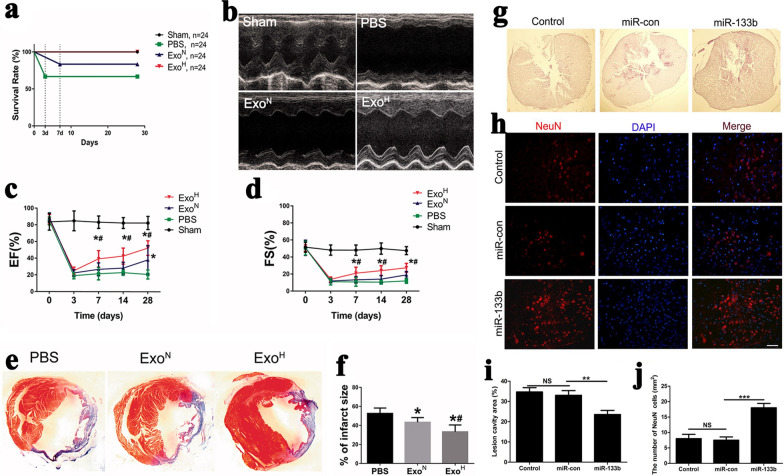
Modulated sEVs for tissue repair/regeneration [26, 78]. **a**–**f** Augmenting cardiac function and ameliorating fibrosis after treated by exosomes derived from MSCs under hypoxia. All images are reproduced with permission from Zhu et al. [78]. Copyright 2018 Taylor & Francis. All rights reserved. **g**–**j** Injection of miR-133b exosomes reduced the lesion volume and preserved NeuN + neurons after spinal cord injury. All images are reproduced with permission from Li et al. [26]. Copyright © 2018 Author(s). All rights reserved

sEVs have shown many advantages in tissue repair and regeneration. In recent years, the applications of stem cells derived sEVs in various damage repair and tissue regeneration have been extensively studied [17]. In different applications, those methods like low oxygen, chemical regulator, biomaterials, or known DNA plasmid transfection could increase the production of sEVs derived from donor cells and enhance the required functions of sEVs. For example, hypoxic pretreatment of CPCs or MSCs not only enhanced sEV production, but also increased the content of specific exosomal components which significantly improved the angiogenesis ability [77–79]. In myocardial infarction mice, after hypoxia-preconditioned MSCs-derived exosome treatment, the infarct size was reduced, and cardiac function was improved obviously (Fig. 7a–f) [78]. The sEVs derived from umbilical cord mesenchymal stem cells (UC-MSCs) stimulated with 3,3′-diindolylmethane (DIM) had higher Wnt 11 content than sEVs derived from normal UC-MSCs [130]. Moreover, compared to untreated UC-MSCs-derived sEVs, sEVs derived from DIM stimulated UC-MSCs had stronger capacity to promote wound healing and skin repairing. Li et al. transfected miR-133b into rat bone marrow derived MSCs through liposomes, upregulating miR-133b in exosomes [26]. It significantly improved the function of exosomes to protect neurons and promote axonal regeneration (Fig. 7g–j) [26]. Xin et al. used lentivirus to transfect miR-133b into bone marrow derived MSCs, upregulating miR-133b in sEVs as well [27]. Injecting these sEVs into brain regulated the gene expression of neurons and astrocytes, promoted the remodeling of neuronal synapses, and accelerated the recovery of nerve function after stroke. Peng et al. found that microenergy acoustic pulse (MAP) promoted rat Schwann cells (SCs) proliferation, neurotropic factor expression and sEV secretion in a dosage response manner, peaking at 100 pulses (0.033 mJ/mm^2^, 1 Hz) [131]. Moreover, those SC-derived sEVs significantly enhanced neurite outgrowth from major pelvic ganglion in vitro. MAP may have utility in the treatment of neurogenic erectile dysfunction by promoting SC-derived sEV production.

### Modulated sEVs for other disease treatment

After years of research and attempts, the application potential of modulated sEVs in other disease treatment has also been continuously explored. The EXOtic device could not only load specific therapeutic nucleic acids into sEVs by transfecting plasmids into donor cells, but also significantly increase the production of sEVs and give them ability to target sEVs [29]. Engineered sEV-producer cells transfected with EXOtic were injected into Parkinson’s model mice to generate sEVs containing catalase mRNA. The sEVs containing catalase mRNA was targeted to the striatum in brain. It reduced neuroinflammation-related factors in brain and relieved the symptoms of Parkinson's disease. Canfrán-Duque et al. found curcumin stimulated the release of sEVs to remove cholesterol that accumulated in the lysosomal cavity [132]. It normalized intracellular lipid homeostasis, thereby helping to relieve the disruption of lipid trafficking induced by antipsychotics. It was also found the sEVs derived from curcumin-treated mouse brain ECs alleviated oxidative stress, reduced the permeability of tight junctions and adherent junction and finally promoted the recovery of the blood–brain barrier in hyperhomocysteinemia [85]. Cui et al. found that sEVs derived from MSCs under hypoxic condition ameliorated cognitive decline by rescuing synaptic dysfunction and regulating inflammatory responses in APP/PS1 mice [133].

## Conclusion and prospects

The sEVs have great potentials in biological medicine field, but their practical applications require a quite high dosage. Meanwhile, for different diseases and different tissue injuries, applying sEVs with different active ingredients and specific biological function is of great significance [17, 22]. Unfortunately, for noncancerous cells, the production of sEVs is severely limited and the biological function of sEVs is varying as cell aging [18, 24]. Therefore, various strategies have been developed in order to realize rapid and precise regulation of the production and function of sEVs derived from required donor cells. Part of these strategies come from the basic research on sEV biogenesis and release, and part of them come from the point of regulating cell behaviors to regulate cell sEV secretion. Besides, it is feasible to load drug and nucleic acid with known therapeutic properties into sEVs by preconditioning donor cells through physical methods, chemical methods, and gene engineering methods.

Among the strategies mentioned above, physical methods and gene engineering methods could more than tenfold increase sEV production according to the reports [29, 97]. However, more intense stimulation and profound impact on cells were caused by gene transfection and high dose physical stimulus. Whether these methods bring about unfavorable consequence on cells and their sEVs is hard to predict. Critical safety assessment of the sEVs produced by stimulating cells with these methods is required before clinical applications of these sEVs. Excessive stimulation on cells to produce sEVs may be harmful to donor cells and the enhancement of sEV production may be unsustainable. Proper stress-inducing conditions, physical mothed, chemical regulator and biomaterials can provide mild stimulus on cells, which could lead to a slight increase (less than 5 times) of sEV yield accordingly [77, 81, 125]. Meanwhile, these mild stimuli can bring sEVs some improved biological function for specific applications, like inflammation suppression, angiogenesis, and tissue repair/regeneration. It may enable sEVs better display the beneficial effects of donor cell, especially stem cells. According to the actual application scenarios, researchers can choose appropriate strategies to obtain increased yield of sEVs which carry the desired therapeutic capability. For example, gene engineering methods and chemical regulators are suitable choices to regulate sEVs for cancer treatment, while physical methods and biomaterial stimulations may be more beneficial to modulate sEVs for tissue repair (Table 4).

**Table 4:** Comparison of different strategies for sEV regulation

Strategy	Advantages	Disadvantages	Proper application scenarios
Gene engineering methods	Abundant regulatory targets Easy to design genetic modification methods based on the biogenesis and release mechanisms of sEVs Easy to load customed nucleic acid	Complicated and expensive Easy to cause unknown mutations in donor cells Low throughput of modulating donor cell	Cancer treatment & gene therapy
Stress-inducing conditions	Convenient operation Capable to enhance sEV yield and strengthen desired biological function High throughput of modulating donor cell	Need to accurately control the stress-inducing conditions Easy to damage donor cells	Cardiovascular disease treatment
Chemical regulators	Convenient operation Capable to enhance sEV yield and strengthen desired biological function High throughput of modulating donor cell	Need to screen chemical regulators from a huge number of chemical molecules Potential cytotoxicity	Cancer treatment
Physical methods	Capable to enhance sEV yield and strengthen desired biological function High throughput of modulating donor cell	Need additional equipment Difficult to accurately control the parameters of physical stimulus Easy to damage donor cells Unclear regulatory mechanism	Tissue repair
Biomaterial stimulations	Capable to enhance sEV yield and strengthen desired biological function Cause no damage to donor cells High safety	Need to prepare various biomaterials with different components and structures Unclear regulatory mechanism	Tissue repair

To achieve sEV mass production and application, appropriate bioreactors for expanding the scale of donor cell culture is also indispensable [134–136]. Therefore, the optimal strategies to regulate the production and biological function of sEVs should be expansive and compatible with bioreactor [136, 137]. So far, large-scale gene transfection is still a tough and high-cost work. Applying hypoxia condition and physical stimulus on cells cultured in the bioreactor are feasible with several available external devices, such as perfusion culture system, ultrasonic equipment, electromagnetic equipment, and atmosphere control device. Chemical regulators and biomaterials are easy and convenient to combine with a bioreactor. Specially, biomaterials can be engineered as scaffolds to mimic the native extracellular matrix structure and functions. In the bioreactor system, it is very promising to develop biomaterial scaffolds as microcarrier. The biomaterial microcarrier may not only provide space for cell attachment and growth but also regulate sEV biogenesis and release. To cooperate with large-scale cell culture and sEV production system, high-throughput and rapid methods for sEV purification and enrichment are also essential, such as tangential flow filtration [135, 138].

In general, current understanding of the biogenesis and release process of sEVs in different types of cells is still insufficient, and the regulation strategies of sEV secretion are not comprehensive and systematic. Mechanism of these regulation strategies and the changes in components and functions of sEVs regulated by these methods still need to be further revealed. In addition to the strategies mentioned above, there are other emerging methods for increasing the production and modulating the biological function of sEVs, such as improving the efficiency of sEVs separation and purification, exosome mimetics produced by extrusion, artificial synthesis of small-sized vesicles with similar function to sEVs and direct loading drugs and nucleic acids into sEVs [103, 136, 139–141]. In the future, if uniform and stable sEVs could be rapidly and liberally produced and the desired therapeutic capability of sEVs could be customized and enhanced, the great potential of sEVs in diseases treatment would be fully developed and the clinical applications of sEV therapy would reach a new level.

## Abbreviations

EVs: Extracellular vesicles
sEVs: Small extracellular vesicles
ESCRT: Endosomal sorting complex required for transport
ILVs: Intraluminal vesicles
MVBs: Multivesicular bodies
HSP: Heat-shock protein
SNARE: Soluble *N*-ethylmaleimide-sensitive fusion attachment protein receptors
EXOtic: Exosomal transfer into cells
MSCs: Mesenchymal stem cells
CPCs: Cardiomyocyte progenitor cells
ECs: Endothelial cells
TGF-β: Transforming growth factor β
Exo ^H^: Exosomes secreted by cells cultured under hypoxic condition
Exo ^N^: Exosomes obtained from cells cultured under normoxic condition
CNP: Cellular nanoporation
SkMCs: Skeletal muscle cells
LIUS: Low-intensity ultrasound
LIPUS: Low-intensity pulsed ultrasound
BMDCs: Bone marrow dendritic cells
MIONs: Magnetic iron oxide nanoparticles
APCs: Antigen-presenting cells
DCs: Dendritic cells
AuNPs: Gold nanoparticles
CaP: Calcium phosphate
BG: Bioglass
EXOmotifs: Short sequence motifs over-represented in miRNAs
PTEN: Orthotopic phosphatase and tensin homologue
UC-MSCs: Umbilical cord mesenchymal stem cells
DIM: 3,3'-Diindolylmethane
SCs: Schwann cells
